# Thermodynamic Insights for Electrochemical Hydrogen Compression with Proton-Conducting Membranes

**DOI:** 10.3390/membranes9070077

**Published:** 2019-07-01

**Authors:** Benjamin L. Kee, David Curran, Huayang Zhu, Robert J. Braun, Steven C. DeCaluwe, Robert J. Kee, Sandrine Ricote

**Affiliations:** Mechanical Engineering, Colorado School of Mines, Golden, CO 80401, USA

**Keywords:** electrochemical compression, proton-conducting membranes, protonic-ceramics, steam reforming

## Abstract

Membrane electrode assemblies (MEA) based on proton-conducting electrolyte membranes offer opportunities for the electrochemical compression of hydrogen. Mechanical hydrogen compression, which is more-mature technology, can suffer from low reliability, noise, and maintenance costs. Proton-conducting electrolyte membranes may be polymers (e.g., Nafion) or protonic-ceramics (e.g., yttrium-doped barium zirconates). Using a thermodynamics-based analysis, the paper explores technology implications for these two membrane types. The operating temperature has a dominant influence on the technology, with polymers needing low-temperature and protonic-ceramics needing elevated temperatures. Polymer membranes usually require pure hydrogen feed streams, but can compress H${}_{2}$ efficiently. Reactors based on protonic-ceramics can effectively integrate steam reforming, hydrogen separation, and electrochemical compression. However, because of the high temperature (e.g., 600 °C) needed to enable viable proton conductivity, the efficiency of protonic-ceramic compression is significantly lower than that of polymer-membrane compression. The thermodynamics analysis suggests significant benefits associated with systems that combine protonic-ceramic reactors to reform fuels and deliver lightly compressed H${}_{2}$ (e.g., 5 bar) to an electrochemical compressor using a polymer electrolyte to compress to very high pressure.

## 1. Introduction

The objective of this paper is to investigate opportunities for producing compressed hydrogen using proton-conducting membranes via electrochemical hydrogen compression (EHC). Two classes of proton-conducting electrolytes are compared. One, based on a polymer electrolyte (e.g., Nafion), operates at low temperature (e.g., <100 °C), usually needs precious-metal charge-transfer catalysts, and requires a pure, moist, H${}_{2}$ feed stream [1]. The other class, based on protonic-ceramic electrolyte membranes (e.g., yttrium-doped barium zirconates, BZY), operates at high temperature (e.g., 600 °C), can use Ni-based catalysts, and can operate on a moist hydrocarbon feed stream [2,3,4,5,6,7,8,9]. In addition to electrochemical compression, both membrane technologies are being developed into fuel cells and electrolyzers [10].

The present paper develops a thermodynamic analysis that predicts comparative process performance. As in all compression technology, compression should be accomplished at temperatures as low as possible and under conditions as close to isothermal as possible. Because the polymer-based systems operate at significantly lower temperatures than the protonic-ceramic systems do, the polymer systems offer inherently higher compression efficiencies. However, the polymer-membrane systems require precious-metal catalysts and pure hydrogen feed streams. Operating at high temperature, protonic-ceramic systems can operate with hydrocarbon fuels and enable internal reforming using Ni-based electrodes. However, because of the relatively low compression efficiencies, protonic-ceramic compressors are likely more viable for relatively low compression ratios.

### 1.1. Current Hydrogen Production

Currently, hydrogen is produced on industrial scales using steam methane reforming (SMR) [11]. Hydrogen is primarily used for ammonia production and petroleum processing [12]. Steam electrolysis and other approaches contribute only a small fraction of the greater than 60 tons of hydrogen that are produced annually. It is reasonable to expect that even greater hydrogen production will be needed as the world trends to hydrogen-based energy economies [13,14,15,16,17]. For current fuel-cell automobiles to achieve ranges comparable to internal combustion engines, fuel-cell vehicles carry hydrogen tanks at 700 bar [18]. To fuel cars at 700 bar, the filling-station storage tanks must be at even higher pressures.

**Figure 1:**
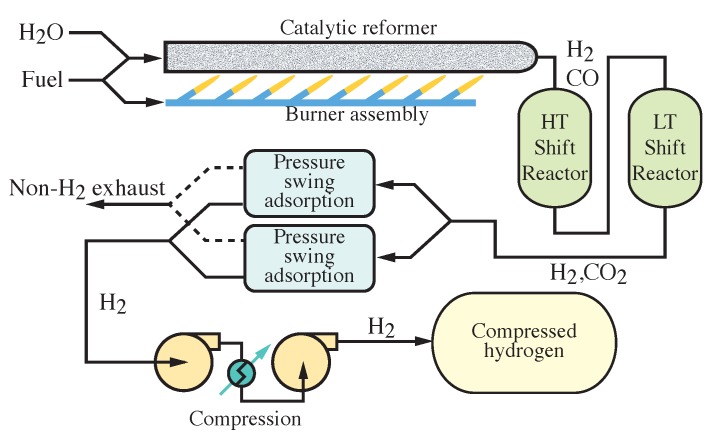
Process flow diagram for a typical industrial process to convert hydrocarbon fuels to compressed hydrogen. The two serial shift reactors operate at high (HT) and low (LT) temperature.

Figure 1 illustrates the three major steps (fuel reforming, separation, and compression) involved in producing compressed hydrogen at the industrial scale. The hydrogen is produced by catalytic steam methane reforming (CH${}_{4}$ + H${}_{2}$O ⇌ 3H${}_{2}$ + CO), which is endothermic and requires temperatures near 600 °C. To further increase H${}_{2}$ yield, the reformer is typically followed by water-gas shift (WGS) reactors (CO + H${}_{2}$O ⇌ H${}_{2}$ + CO${}_{2}$) where the carbon monoxide is further oxidized to produce H${}_{2}$ and CO${}_{2}$ in the presence of excess steam [19]. Even with the shift reactors, the hydrogen may not be sufficiently pure. Hydrogen separation can be accomplished using pressure-swing-adsorber (PSA) technology. Palladium membranes may also be used for hydrogen separation, although they can be susceptible to CO poisoning [20,21,22,23]. Compression is usually accomplished with reciprocating or rotating mechanical compression [13].

The overall H${}_{2}$-production process suffers some inherent inefficiencies due to the large number of process steps [19]. Hydrogen production by SMR generally requires approximately 46 kWh kg${}_{H_{2}}^{-1}$ at 72% efficiency [17]. The efficiency can easily vary from 60 to 80% based on specific circumstances such as operating conditions, efficiency definition, and heat recovery options, however the majority of the efficiency losses occur in the reforming [24,25,26,27]. A fraction of the fuel must be burned to heat the catalytic reformer to support the endothermic reforming. The PSA process requires multiple vessels to alternate between adsorbing and desorbing contaminants. Pressurizing and purging the vessels demands additional overhead. Nordio et al. [28] compared a low temperature electrochemical hydrogen compressor to PSA technology and showed that electrochemical hydrogen compression was more worthwhile at small scales and elevated outlet hydrogen pressure. Mechanical compressors have significant maintenance costs, and can be noisy. The maintenance to avoid leaks has been estimated to be 90% of the overall maintenance costs [1,29]. While mechanical compressors may have problems, they are mature and commercially available [17].

### 1.2. Proton-Conducting Membranes

Electrochemical hydrogen compression offers a potentially viable and cost-competitive alternative to mechanical compression. Operation requires low-voltage, direct-current, electrical energy to polarize the proton-conducting membrane. As such, integration with renewable sources such as photovoltaics may be attractive. Current electrochemical compression devices are based on polymer electrolyte membranes (PEM) that operate around 100 °C. PEM membranes are relatively mature, especially for fuel cells [1]. PEM-based electrochemical compressors operate at efficiencies that are comparable to the best mechanical compressors [17]. In principle, they can be more reliable and do not suffer problems such as oil contamination [17,28]. Nevertheless, there are practical considerations, including hydrogen back-diffusion, Ohmic (resistive) losses, inlet purity, water management, catalyst performance, and material costs [17].

Compared to polymer membranes, protonic ceramics are far less mature. The proton-ceramic membranes are doped perovskites, such as yttrium-doped barium zirconates and cerates [10,30,31,32,33,34]. While they are called proton conductors, these materials in fact are mixed ionic-electronic conductors (MIEC). The most studied protonic ceramics are yttrium-doped solid solutions of barium cerate/zirconate, which combine stability and satisfactory protonic conductivity [35,36]. These materials typically have three mobile charged defects—protons OH${}_{O}^{•}$, oxygen vacancies $V_{O}^{••}$, and small polarons $O_{O}^{•}$ [31]. In operation for electrochemical compression, the proton transport is dominant. Nevertheless, there can be some electronic leakage via small polarons [33].

Protonic defects OH${}_{O}^{•}$ may be incorporated into the perovskite lattice via steam dissociation or directly from H${}_{2}$ as

(1) $$H_{2}O\left(g\right)+V_{O}^{••}+O_{O}^{\times }⇌2{\mathrm{OH}}_{O}^{•},$$

(2) $$\frac{1}{2}H_{2}\left(g\right)+O_{O}^{•}⇌{\mathrm{OH}}_{O}^{•}.$$

Once incorporated in the lattice, proton transport proceeds via the Grotthuss mechanism [37,38].

Most applications using protonic ceramics require operation within a furnace to maintain the membrane at operating temperature (500–800 °C). During polarization, Ohmic and Faradaic heating represent energy sources that help to heat the membrane [39].

## 2. Compression Thermodynamics

The present thermodynamics study is based on some significant idealizations and assumptions. Thus, the results should be understood as providing quantitative insights, but subject to limiting-case approximations. The study considers isothermal (constant temperature) and isentropic (constant entropy) compression. By definition, isothermal compression operates at constant temperature, but must reject a large amount of heat. Isentropic compression produces no entropy, but the temperature increases greatly. Any real process is bounded by these limiting cases.

At sufficiently high pressures, the ideal-gas law becomes inaccurate. The present analysis compares performance using ideal-gas and real-gas Helmholtz equations of state. Todd et al. [40] investigated the thermodynamics of high-temperature and high-pressure water electrolysis using high-fidelity non-ideal equations of state.

**Figure 2:**
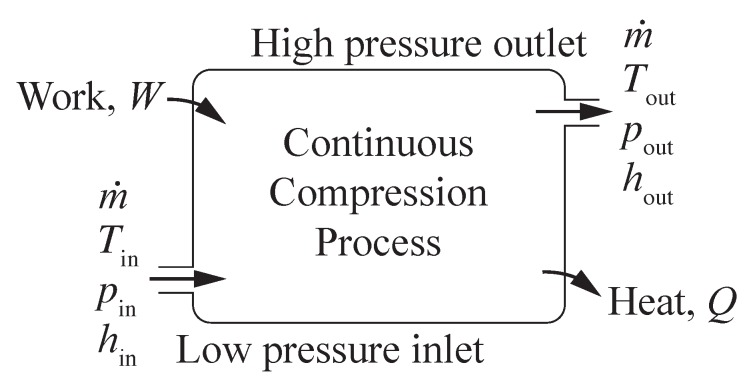
Hydrogen flowing through a compressor, exiting at increased pressure due to work added and heat rejected.

Figure 2 illustrates a generic, steady-state, compression process, with hydrogen entering at low pressure and exiting at high pressure. Flow enters the compressor at temperature $T_{\mathrm{in}}$, pressure $p_{\mathrm{in}}$, and mass flow rate $\dot{m}$. The outlet stream of hydrogen exits at temperature $T_{\mathrm{out}}$, pressure $p_{\mathrm{out}}>p_{\mathrm{in}}$, and the same mass flow rate $\dot{m}$ (kg s${}^{-1}$). The energy associated with the hydrogen compression is represented through enthalpy *h* (kJ kg${}^{-1}$). Work $\dot{W}$ (kW) is done on the system to increase the pressure. Heat $\dot{Q}$ (kW) is transferred from the process to the environment. The steady-state energy balance for the open flowing system may be represented as

(3) $$\dot{W}+\dot{m}h_{\mathrm{in}}=\dot{Q}+\dot{m}h_{\mathrm{out}}.$$

The energy balance can be normalized by the mass flow rate, where *w* (kJ kg${}^{-1}$) is the intensive work done on the system and *q* (kJ kg${}^{-1}$) is the intensive heat transfer out of the system. Assuming the idealization of reversible heat transfer, the second law of thermodynamics provides that

(4) w=∆h−T∆s.

The specific work *w* may be evaluated as w=∫vdp, where $v=\left(R_{\mathrm{gas}}T\right)/\left(\bar{M}p\right)$ is the specific volume, $R_{\mathrm{gas}}=8.314$ J mol${}^{-1}$ K${}^{-1}$, and $\bar{M}$ is the molecular weight. For equations of state other than ideal gas, analytically integrating vdp may be difficult. Thus, evaluating the work as a function of end states, as in Equation (4), can be advantageous.

### 2.1. Isothermal Compression

Assuming isothermal operation, because *h* is a function of temperature alone for an ideal gas, Equation (4) reveals that the work is only a function entropy alone. For an ideal gas, the vdp integral can be evaluated in terms of pressure end states as

(5) $$w=\int_{\mathrm{in}}^{\mathrm{out}}vdp=\frac{R_{\mathrm{gas}}T}{\bar{M}}\ln\left(\frac{p_{\mathrm{out}}}{p_{\mathrm{in}}}\right).$$

Real gas properties are evaluated with Engineering Equation Solver (http://www.fchart.com/ees/) using the Helmholtz equation of state as described by Leachman et al. [41]. The compression work is evaluated in terms of the enthalpy and entropy end states. In practical applications, hydrogen may be compressed to pressures as high as 1000 bar. At these pressures, the ideal-gas equation of state is inaccurate.

**Figure 3:**
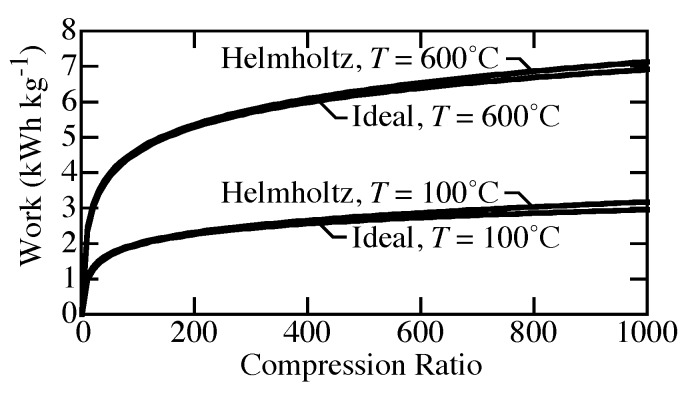
Comparison of compression work for real-gas and ideal-gas equations of state from 1 to 1000 bar at temperatures of 100 °C and 600 °C.

Figure 3 compares the specific work needed to compress H${}_{2}$ isothermally from atmospheric pressure to pressures up to 1000 bar. The specific work needed at 100 °C is much lower than that needed to compress at 600 °C. Real-gas effects at high pressure increase the needed work compared to ideal-gas behavior. However, although perhaps not negligible, the real-gas effects are not great. At 1000 bar, the difference between ideal-gas and real-gas equations of state (EOS) reaches about 0.2 kWh kg${}^{-1}$.

**Figure 4:**
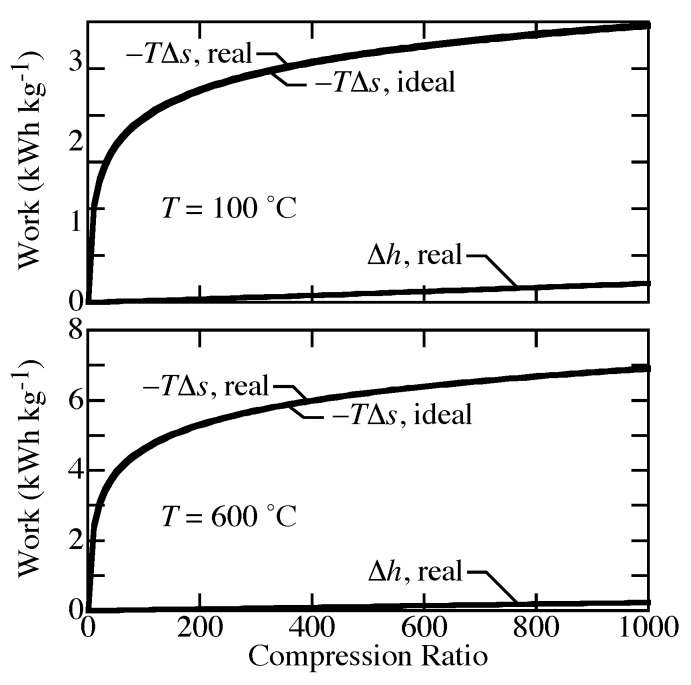
Comparison of entropy and enthalpy changes for real-gas and ideal-gas equations of state for compression from 1 bar to 1000 bar at 100 °C and 600 °C.

The differences between ideal-gas and real-gas effects on compression work can be described by the relative differences in entropy T∆s and enthalpy ∆h. Figure 4 shows that T∆s is nearly the same for both equations of state. The differences in work are caused primarily by ∆h. For an ideal gas, the enthalpy depends on temperature alone, so ∆h=0 for isothermal compression. However, real-gas behavior includes the effect of pressure on enthalpy, which directly affects the work.

To maintain isothermal compression, a great deal of heat must be removed. In fact, most of the work put into the system leaves as heat. As a practical matter, removing heat effectively can be technologically challenging. Interstage cooling is often used to approximate isothermal conditions.

### 2.2. Isentropic Compression

**Figure 5:**
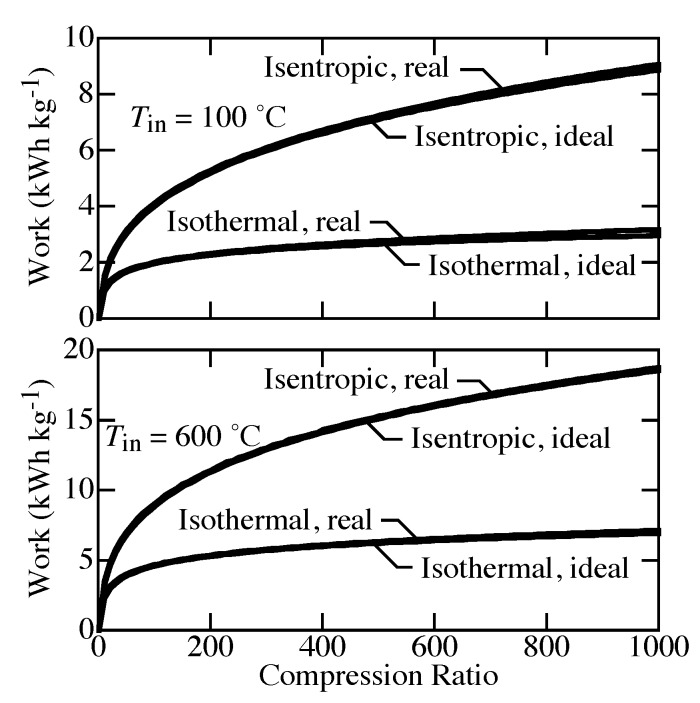
Comparison of isothermal and isentropic compression from 1 to 1000 bar at inlet temperature of 100 and 600 °C. The fitting parameters used for the Helmholtz EOS are valid for temperatures below 727 °C [41].

Isentropic compression is accomplished conceptually by assuming a perfectly reversible and insulated process. Under such conditions, the compression work may be represented as

(6) $$w=∆h=\int_{\mathrm{in}}^{\mathrm{out}}v\left(s,p\right)dp,$$

where the specific volume v(s,p) is evaluated at the inlet entropy and pressure. Figure 5 shows differences between the ideal isentropic and isothermal compression. The real-gas EOS plays only a small role. The compression work for isentropic compression is much larger than it is for isothermal compression. The work depends strongly on temperature. For isentropic compression, the temperature must increase to keep the process adiabatic.

**Figure 6:**
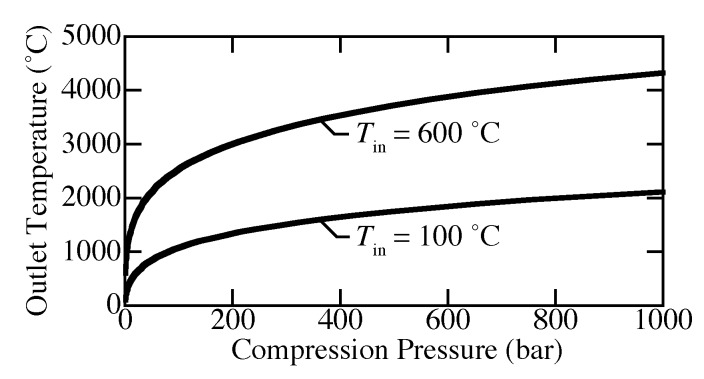
Exit temperature for isentropic compression from 1 to 1000 bar at inlet temperatures of 100 °C and 600 °C. Recommended use of the Helmholtz EOS is for temperatures below 727 °C [41].

Figure 6 shows the outlet temperature as a function of compression pressure where the inlet is 100 °C or 600 °C and 1 bar. The ideal-gas equation of state and the Helmholtz equation of state predict nearly the same outlet temperature (overlapping in the figure). Isentropic compression causes unreasonable increases in temperature. For compression from 1 to 200 bar, the outlet temperature already exceeds 1000 °C for inlet temperature of both 100 °C and 600 °C. In other words, the ideal isentropic compression is practically unachievable.

## 3. Electrochemical Compression

**Figure 7:**
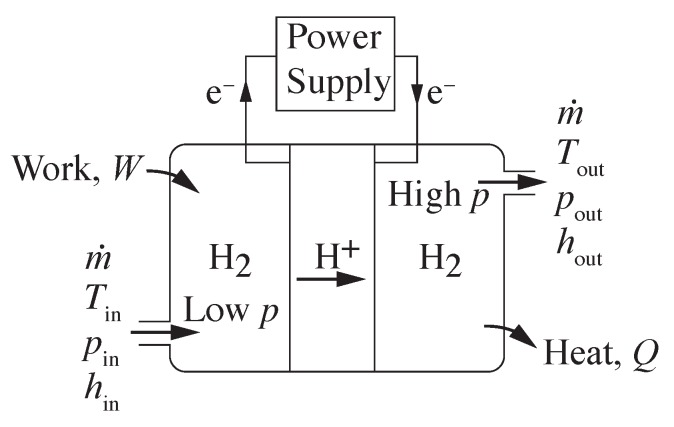
Compression across a proton conducting membrane.

Figure 7 illustrates an electrochemical hydrogen compressor using a polarized proton conducting membrane. The protons crossing the membrane must be balanced by the electrical current supplied by an external circuit. The flow of hydrogen through a proton conducting compression system must be equal to the net transport of protons through the membrane. The required electrical current *I* may be evaluated as

(7) $$I=\frac{n\dot{m}F}{M_{H_{2}}}$$

where $\dot{m}$ is the rate of hydrogen being compressed, n=2 is the number of electrons per mole of hydrogen, $F=96485\times 10^{3}$ C kmol${}^{-1}$ is the Faraday constant, and $M_{H_{2}}$ = 2.016 kg kmol${}^{-1}$ is the molecular weight of hydrogen. The cell voltage may be evaluated as

(8) $$E_{\mathrm{cell}}=E_{\mathrm{OCV}}+IR,$$

where, assuming a pure proton conducting membrane, $E_{\mathrm{OCV}}$ is the open-circuit voltage (OCV) that may be evaluated as

(9) $$E_{\mathrm{OCV}}=\frac{R_{\mathrm{gas}}T}{nF}\ln\left(\frac{p_{\mathrm{out}}}{p_{\mathrm{in}}}\right).$$

**Figure 8:**
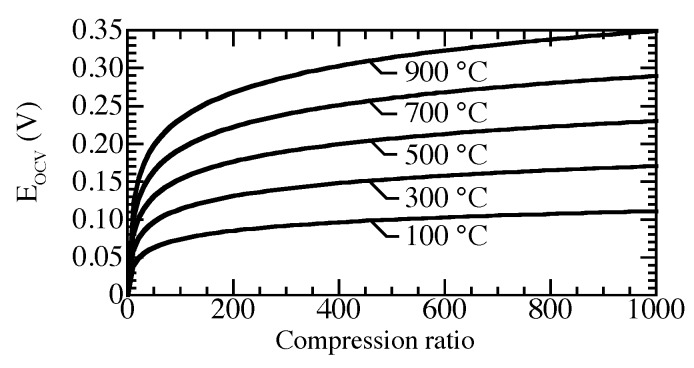
Open-circuit voltage, compressing from 1 bar across a proton-conducting membrane for pressure ratios between 1 and 1000, and temperatures between 100 °C and 900 °C.

The open-circuit voltage depends on the compression ratio and the operating temperature. Figure 8 shows that the open circuit voltage increases as the temperature or compression ratio increases. Membranes operating at high temperature require higher voltage to reach the same compression ratio as membranes operating at low temperature.

The proton resistance across the membrane can be represented empirically in terms of an area specific resistance (ASR),

(10) $$R=\frac{\mathrm{ASR}}{A_{\mathrm{mem}}},$$

where $A_{\mathrm{mem}}$ is the membrane area. Proton-conducting membranes typically have ASR ranging between 0.1 Ω cm${}^{2}$ and 1.0 Ω cm${}^{2}$ [28,35,36,42].

The voltage efficiency of electrochemical compression may be defined as

(11) $$\varepsilon =\frac{E_{\mathrm{OCV}}}{E_{\mathrm{OCV}}+IR},$$

where operating at open-circuit voltage yields 100% voltage efficiency. However, at open-circuit voltage no compression is being accomplished.

The compression power can be evaluated as

(12) $$P_{\mathrm{cell}}=E_{\mathrm{cell}}I.$$

By substituting Equations (7), (8), and (10) into Equation (12) the gravimetric power density or specific energy (kWh kg${}^{-1}$) can be evaluated as

(13) $$\frac{P_{\mathrm{cell}}}{\dot{m}}=\mathrm{ASR}{\left(\frac{nF}{M_{H_{2}}}\right)}^{2}\frac{\dot{m}}{A_{\mathrm{mem}}}+\frac{nF}{M_{H_{2}}}E_{\mathrm{OCV}}.$$

The compression specific energy (kWh per kg of H${}_{2}$ compressed) is directly proportional to the membrane ASR, the hydrogen mass flow rate $\dot{m}$, and inversely proportional to the membrane area. The effects of temperature and pressure enter via $E_{\mathrm{OCV}}$. ASR also depends on temperature and pressure, but those underlying physical processes are not directly incorporated into the present analysis. Rather, ASR is used as a parameter.

**Figure 9:**
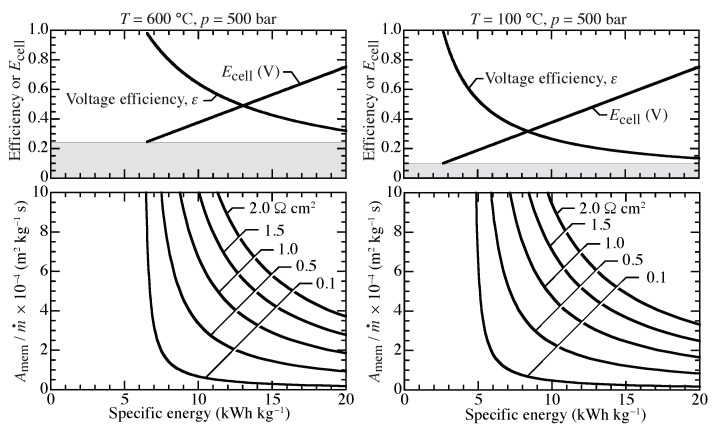
Isothermal electrochemical compression from 1 to 500 bar across a proton conducting membrane at 600 and 100 °C for area specific resistance (ASR) between 0.1 Ω cm${}^{2}$ and 2 Ω cm${}^{2}$.

Assuming a compression ratio of 500, Figure 9 graphically represents Equation (13), showing relationships between cell voltage, voltage efficiency, and required membrane area as functions of specific energy. At 100 °C, the conditions represent an isothermal PEM compressor. The 600 °C case represents an isothermal protonic-ceramic compressor. The shaded region indicates the lower bound for operating voltage, or the OCV. Operating below the OCV allows hydrogen to be transported from the high pressure side to the low pressure side. The OCV is higher at higher temperature, causing the minimum specific energy to be higher at higher temperature.

The lower graphs of Figure 9 can be used to evaluate the membrane area required to compress from 1 bar to 500 bar at two temperatures. Broadly speaking, practical compression energies are on the order of 10 kWh per kg of H${}_{2}$ compressed. As is evident from Figure 9, higher ASR membranes require significantly greater membrane areas, especially at lower compression energies. The operating cell voltage is a function of the specific energy, but is independent of membrane ASR. Voltage efficiency depends very weakly on ASR.

A typical polymer electrolyte membrane has an ASR on the order of 0.1 Ω cm${}^{2}$ [28,42]. For a 25 μm thick membrane with conductivity of 4 mS cm${}^{-1}$ [35,36], a typical protonic-ceramic membrane has an ASR on the order of 1 Ω cm${}^{2}$. The membrane area can vary by an order of magnitude for ranges of ASRs considered here. Lower ASR membranes consistently outperform higher ASR membranes in the sense that less energy and area are required for comparable compression. For an ASR of 0.1 Ω cm${}^{2}$ and a specific energy of 10 kWh kg${}^{-1}$ at 600 °C, a specific membrane area of approximately $A_{\mathrm{mem}}/\dot{m}=1\times 10^{4}$ m${}^{2}$ kg${}^{-1}$ s is required. In this example, increasing the ASR to 1.0 Ω cm${}^{2}$ requires approximately 7.5 times more membrane area.

High ASRs lead to large compression power requirements. High hydrogen mass flow rates demand high power for compression. The lowest possible power requirement occurs at OCV. However, near OCV the compression rates are small and the membrane areas are large. As membrane area decreases, more power is required to achieve comparable compression rates.

### Case Study—Required Membrane Area for Low Compression Ratio

**Figure 10:**
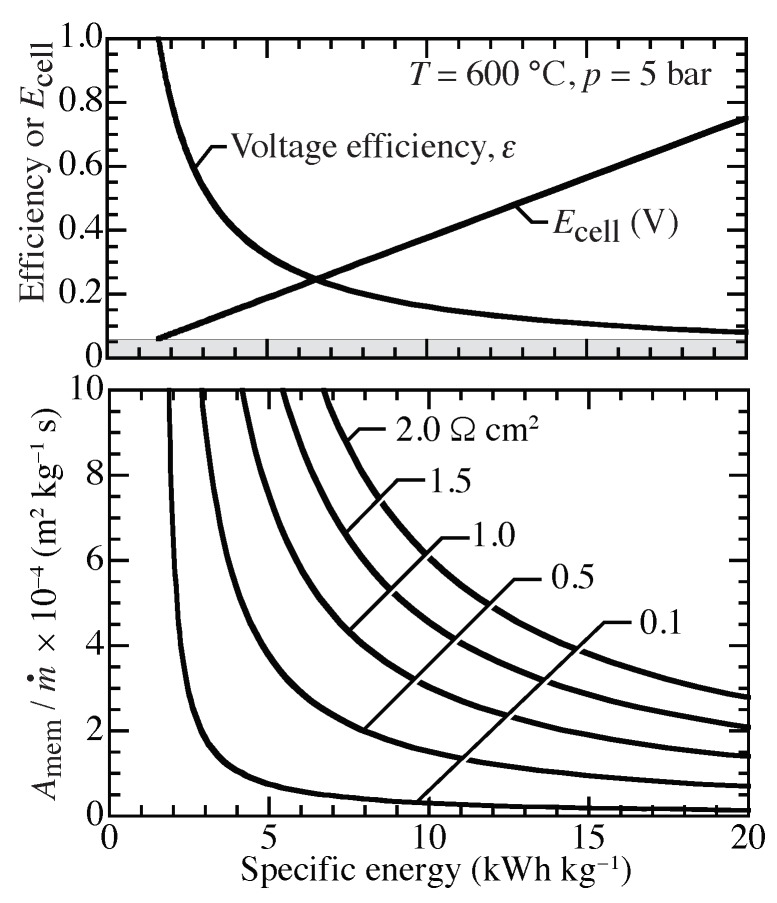
Isothermal electrochemical compression from 1 to 5 bar across a proton-conducting membrane at 600 °C with ASR between 0.1 Ω cm${}^{2}$ and 2 Ω cm${}^{2}$.

Assuming isothermal compression from 1 bar to 5 bar at 600 °C, Figure 10 shows the needed membrane area as a function of ASR and compression specific energy. For example, with a specific compression energy of approximately 10 kWh kg${}^{-1}$ and ASR=0.5 Ω cm${}^{2}$, Figure 10 indicates a required specific membrane area of $A_{\mathrm{mem}}/\dot{m}\approx 1.5\times 10^{4}$ m${}^{2}$ kg${}^{-1}$ s. For a target of 10 kg per day (24 h) at 5 bar, the required membrane area would be approximately 1.74 m${}^{2}$. The membrane area could be implemented in planar, bipolar stacks. Assuming 100 cm${}^{2}$ planar membranes, a 174 layer stack is needed. Assuming a nominal 3 mm per layer, such a stack would occupy a volume of approximately 10 × 10 × 52 cm${}^{3}$. Decreasing the ASR or reducing the compression temperature would greatly decrease the needed membrane area. Potential pathways for reducing ASR include decreasing membrane thickness and improving electrodes.

## 4. Steam Reforming

Steam reforming is a highly endothermic catalytic process (e.g., Ni catalyst) that is effective at temperatures around 600 °C. Thus, the reforming temperatures are compatible with the temperatures used for protonic-ceramic membranes. Combining steam reforming with protonic-ceramic compression can synergistically and beneficially integrate three processes — fuel reforming, hydrogen separation, and hydrogen compression [2]. The thermal energy needed to support the reforming endotherm can be supplied by the heating associated with proton transport through the membrane and heat of compression. The integrated process serves to assist maintaining process temperature and achieving isothermal compression.

The combination of steam-methane reforming (SMR) and water-gas shift (WGS) may be expressed globally as

(14) $${\mathrm{CH}}_{4}+2H_{2}O⇌4H_{2}+{\mathrm{CO}}_{2}.$$

The net energy required by SMR process includes heating the reactants (sensible and latent heat) and the enthalpy of reaction as

(15) $$Q_{\mathrm{SMR}}=∆H_{\mathrm{SMR}}+∆H_{{\mathrm{CH}}_{4},2H_{2}O\left(25{}^{\circ}C\to 600{}^{\circ}C\right)}.$$

The heat of reaction may be evaluated by assuming full conversion according to the global reaction (Equation (14)). Assume that the feed stream is heated from 25 °C to 600 °C. The heat of reaction for steam-methane reforming at 600 °C is

(16) $$∆H_{\mathrm{SMR}}=46.853\frac{\mathrm{kJ}}{\mathrm{mol}\;H_{2}}=6.46\frac{\mathrm{kWh}}{\mathrm{kg}\;H_{2}}.$$

Energy is required to heat the reactants from ambient conditions to reactor operating conditions. In the case of H${}_{2}$O, the energy requirement must consider the latent heat of vaporization as

(17) $$∆H_{{\mathrm{CH}}_{4},2H_{2}O}\left(25{}^{\circ}C\to 600{}^{\circ}C\right)=39.75\frac{\mathrm{kJ}}{\mathrm{mol}\;H_{2}}=5.5\frac{\mathrm{kWh}}{\mathrm{kg}\;H_{2}}.$$

The global SMR reaction (Equation (14)) is written with the stoichiometric amount of H${}_{2}$O. However, as a practical matter, excess H${}_{2}$O is needed to avoid carbon deposits on the Ni catalysts [43]. Typically, steam-carbon ratios of S/C = 2.5 or greater are needed to mitigate catalyst fouling.

For methane steam reforming, the coke-free chemistry may be represented globally as

(18) $${\mathrm{CH}}_{4}+3H_{2}O⇌4H_{2}+{\mathrm{CO}}_{2}+H_{2}O.$$

In other words, the feed stream includes an additional mole of H${}_{2}$O, which must be elevated to the reactor process temperature. Considering S/C = 3.0,

(19) $$∆H_{{\mathrm{CH}}_{4},3H_{2}O}\left(25{}^{\circ}C\to 600{}^{\circ}C\right)=56.09\frac{\mathrm{kJ}}{\mathrm{mol}\;H_{2}}=7.73\frac{\mathrm{kWh}}{\mathrm{kg}\;H_{2}}.$$

Electrochemical compression through the protonic-ceramic membrane assembly produces heat, owing to Ohmic and Faradaic processes. Ohmic heat is associated with resistance to proton transport within the protonic-ceramic membrane. Faradaic heat is the result of polarization (overpotentials) associated with the charge-transfer process to incorporate protons into the membrane. The net area specific resistance (ASR), which empirically represents all these processes, depends on numerous design and fabrication details. The ASR for the yttrium-doped barium zirconates/cerates materials is on the order of ASR ≈ 1 Ω cm${}^{2}$.

Assuming ASR=1 Ω cm${}^{2}$ and an electrical current density of *i* = 1 A cm${}^{-2}$, the heat flux (per membrane area) owing to membrane polarization and proton flux is $Q_{\mathrm{MEA}}^{\prime \prime }\approx 1$ W cm${}^{-2}$. Further assuming a Faradaic efficiency of approximately 100% (i.e., each electron supplied delivers one proton through the membrane), the net heat production can be evaluated as

(20) $$Q_{\mathrm{polarization}}=28.2\frac{\mathrm{kWh}}{\mathrm{kg}\;H_{2}}.$$

If the membrane resistance were reduced to ASR=0.5 Ω cm${}^{2}$, then the net heat would be reduced to

(21) $$Q_{\mathrm{polarization}}=14.9\frac{\mathrm{kWh}}{\mathrm{kg}\;H_{2}}.$$

Consider a methane feed stream with S/C = 3.0. The net heat required to support the SMR is

(22) $$Q_{\mathrm{SMR}}=∆H_{\mathrm{SMR}}+∆H_{{\mathrm{CH}}_{4},3H_{2}O}=6.46+7.73=14.19\frac{\mathrm{kWh}}{\mathrm{kg}\;H_{2}}.$$

Thus, the system is approximately thermal-neutral when ASR=0.5 Ω cm${}^{2}$.

## 5. Process Integration and Staging

As the foregoing analysis shows, there are pros and cons associated with the low-temperature (polymer based) and the high-temperature (protonic-ceramic based) hydrogen compression. A combination of the two could perform better than either one alone. The thermodynamic analysis makes clear that low-temperature isothermal compression provides the highest compression efficiency. However, using polymer membranes at low temperature requires a pure H${}_{2}$ feed stream. At the commercial scale, such hydrogen is produced and purified in large-scale natural-gas reforming facilities. Delivering the pure H${}_{2}$ to distributed compression stations requires handling and transportation costs.

High-temperature compression is relatively inefficient, but offers other potential advantages. Assuming electricity and natural gas are readily available at distributed locations, the protonic-ceramic compressors could be used for integrated reforming, separation, and first-stage compression. The pure, lightly compressed H${}_{2}$ could then be delivered to a polymer-based compressor to achieve high-pressure compression. Assume, for example, that the protonic-ceramic compressor delivers 5-bar H${}_{2}$. Assume further that the polymer-based compressor is to deliver 1000 bar H${}_{2}$. With a 5-bar feed stream (compared to 1 bar), the compression ratio for the high-pressure compressor is reduced from 1000/1 to 1000/5=200. Reducing the compression ratio by a factor of five can greatly increase the efficiency of the high-pressure compressor.

Integrating protonic-ceramic and polymer-based compression technology offers some potentially attractive benefits. In small-scale, distributed, facilities, such as filling stations, the feed streams can be pipeline natural gas and electricity. The protonic-ceramic unit delivers lightly compressed H${}_{2}$ to the polymer-based compressor, which compresses pure H${}_{2}$ to very high pressure. The integrated solid-state system would be efficient and quiet. The need to transport compressed hydrogen from central reforming plants would be eliminated.

Recently, Corgnale et al. [1,44] modeled an electrochemical hydrogen compressor upstream of a metal-hydride compressor, which proved to be a cost-effective compression process. The staged compressors enabled the metal hydride-compressor to operate with a lower compression ratio. The waste heat in the upstream compressor was diverted to the thermal metal-hydride compressor, which further improved efficiency.

## 6. Summary and Conclusions

This paper develops a thermodynamics analysis that serves as a basis for comparing low-temperature (polymer-based) electrochemical H${}_{2}$ compression with high-temperature (protonic-ceramic) electrochemical compression. While the ideal thermodynamics assumptions neglect many practical engineering issues, the limiting-case results provide great quantitative insight that can guide technology development. Combining the best features, polymer-based and protonic-ceramic compressors may offer pathways to cost-competitive, commercially viable, distributed, hydrogen-compression technology.
